# Energy, Entropy, Constraints, and Creativity in Economic Growth and Crises

**DOI:** 10.3390/e22101156

**Published:** 2020-10-14

**Authors:** Reiner Kümmel, Dietmar Lindenberger

**Affiliations:** 1 Institute for Theoretical Physics und Astrophysics, University of Würzburg, D-97074 Würzburg, Germany; 2 Institute of Energy Economics, University of Cologne, D-50827 Cologne, Germany; dietmar.lindenberger@uni-koeln.de

**Keywords:** energy, economic growth, output elasticities, entropy production, emissions, optimization

## Abstract

The neoclassical mainstream theory of economic growth does not care about the First and the Second Law of Thermodynamics. It usually considers only capital and labor as the factors that produce the wealth of modern industrial economies. If energy is taken into account as a factor of production, its economic weight, that is its output elasticity, is assigned a meager magnitude of roughly 5 percent, according to the neoclassical cost-share theorem. Because of that, neoclassical economics has the problems of the “Solow Residual”, which is the big difference between observed and computed economic growth, and of the failure to explain the economic recessions since World War 2 by the variations of the production factors. Having recalled these problems, we point out that technological constraints on factor combinations have been overlooked in the derivation of the cost-share theorem. Biophysical analyses of economic growth that disregard this theorem and mend the neoclassical deficiencies are sketched. They show that energy’s output elasticity is much larger than its cost share and elucidate the existence of bidirectional causality between energy conversion and economic growth. This helps to understand how economic crises have been triggered and overcome by supply-side and demand-side actions. Human creativity changes the state of economic systems. We discuss the challenges to it by the risks from politics and markets in conjunction with energy sources and technologies, and by the constraints that the emissions of particles and heat from entropy production impose on industrial growth in the biosphere.

## 1. Introduction

Seventy-five years ago Nazi-Germany collapsed. The allied soldiers who liberated the concentration camps, and the camps where more than two million Soviet prisoners of war were starved to death, shocked the world by the documentations of the atrocities commited by a member of European civilization. After unconditional surrender on 8 May 1945, Germany was left with devastated cities, a shattered economy and moral misery.

The rivalry of economic systems and the fortunes of political change saved Germans from more than the usual revenge by the winners of a war. This was especially true for the ones in the west zones as established by the ruling of the Yalta and Potsdam conferences. The antagonism between capitalist market economics of the western occupying powers, who administered what became the Federal Republic of Germany (FRG), and socialist planned economics of the Soviet Union, who occupied what became the German Democratic Republic (GDR), turned allies into adversaries. Tensions between them were enhanced by the Korean War 1950–1953. To strengthen the western camp the FRG was allowed to benefit from the Marshall Plan [1]. Via this European Recovery Program the USA transferred 13.12 billion dollars (corresponding to 139 billion dollars today) between 1948 and 1952 to war-torn Europe. In contrast, the industrial capital goods of the GDR were transferred to the Soviet Union as reparations.

The so-called “economic miracle” of the FRG, which started in 1949 with the currency reform that brought the Deutsche Mark (DM), was based on a growing capital stock, rebuilt and modernized by skilled labor, and cheap oil from the newly discovered oil-fields in the Middle East, Indonesia and the Americas: Between 1950 and 1970 the price of 1 barrel of crude oil on the world market had fallen from about 20 to 12 US${\$}_{2014}$, and economic growth in the western industrialized democracies was up to 7% annually.

Complementing the retrospect on German crash and recovery by the following tale from a physicist [2] shall indicate the limits-to-growth reason that enticed him and other people outside economics to start thinking about economic growth: “Having experienced how industrializiation improves life while I grew up in postwar Germany and then did physics research at the University of Illinois, I joined a project of scientific cooperation between the FRG and the Republic of Colombia. My task was to participate in the development of a master program in physics at the Universidad del Valle. My excellent Colombian colleagues considered the formation of good physicists and engineers as one prerequisite for progress in the industrialization of a, in many aspects, still agrarian society. Right in the beginning they asked me to teach thermodynamics. ‘Anything but this. Thermodynamics is boring’, I objected. ‘Read Reif’s book *Statistical and Thermal Physics*’ [3], they suggested. I did—and for the first time I really understood entropy. When two years later *The Limits to Growth* was published [4], I was deeply shocked. I told my Colombian students that the world would run into trouble because of the Second Law of Thermodynamics, if the developing countries would follow the path of industrialization Europe and the USA have treaded so far—At the celebration of ‘50 years Physics Department of the Universidad del Valle’ in 2013, some of my former students, now physics professors, told me that they well remember how much I had been shocked.—After three unforgettable years in Colombia I returned to Germany. After having settled at the Julius-Maximilians-Universität I got in touch with economists, and in addition to teaching theoretical physics and continuing research in solid state theory I offered courses on thermodynamics and economics. Good students joined research in this field, and experienced economists helped.”

Mostly in plain language, the present article presents a synopsis of the resulting studies on energy and entropy in economic growth. It includes an outlook on options of how to deal with the crises ahead. The review is limited in the extent it covers the literature on energy economics. More on that can be learned from, e.g., Eichhorn et al. [5], Ayres and Warr [6], Hall and Klitgaard [7], Herrmann-Pillat [8], and Ayres [9]. The mathematics, on which our principal findings are based, is packed in an appendix.

## 2. Basic Physics

Whenever something happens, energy is converted and entropy is produced. This summarizes the First and the Second Law of Thermodynamics. More precisely, the First Law on the conservation of energy says that *energy* consists of the never changing sum of *exergy* and *anergy*. Exergy (with *x*) is the valuable part of energy, which can be converted into any form of work that is needed to cause a change, and anergy is the useless part of energy, e.g., heat dumped into the environment. Primary energy such as solar radiation, water power, and—in principle, at sufficiently high process temperatures—fossil and nuclear fuels as well, are 100 percent exergy. The Second Law on the increase of entropy—which is the physical measure of disorder—states that irreversible processes produce entropy. All processes that are not infinitely slow are irreversible. They are triggered by removals of constraints.

Energy-converting activities in natural and economic systems are irreversible. Their entropy production involves heat and particle emissions and destroys exergy. Furthermore, if their impact on the biosphere cannot be balanced by thermal radiation into space and processes activated by the exergy radiated from the Sun to Earth, the living species and their societies face problems of adaptation to environmental changes. In principle, pollution by particles such as SO${}_{2}$, NO${}_{x}$, dust, CO${}_{2}$, and by radioactive waste as well, can be mitigated by appropriate removal techniques and sufficient exergy inputs [10] (Section 3.6). However, even if the emissions of carbon dioxide and other infrared-active trace gases can be curbed so drastically that the anthropogenic greenhouse effect need not worry us any longer, an increasing use of energy from earth-internal sources will cause considerable climate changes, once the heat barrier at about $3\times 10^{14}$ Watts (W) of anthropogenic waste-heat emissions will be surpassed. In 2018, global primary energy consumption was $1.75\times 10^{13}$ W, and the power of solar radiation received by Earth is $1.2\times 10^{17}$ W. [11]

Nicholas Georgescu-Roegen was the first economist to point out the importance of entropy for economic and social evolution in his seminal book *The Entropy Law and the Economic Process* [12]. It stimulated new research on thermodynamics and economics [13,14,15,16]. However, claiming to have discovered a “fourth law of thermodynamics” on the dissipation of matter [17,18] he had created some confusion. This was resolved, when it became clear that the dissipation of matter is included in the Second Law of Thermodynamics [19] via the particle-current-density terms, which are one component of the non-negative density of entropy production derived in non-equilibrium thermodynamics [20]; see also [10] (p. 154ff) and [21].

The *empirical* laws on energy conservation and entropy production are the most powerful laws of nature. Any theory that is against them is doomed to failure.

## 3. Wealth Production and Growth in Conventional Economics

### 3.1. Concepts of Agrarian Society

In 1776, Adam Smith’s “The Wealth of Nations” was published, James Watt’s first steam engines were installed in commercial enterprises, and the “Declaration of Independence” was approved by the Second Continental Congress in Philadelphia. “The Wealth of Nations” founded market economics, the steam engine triggered the industrial revolution, and the “Declaration of Independence” proclaimed the human rights, among them “life, liberty, and the pursuit of happiness.” The human rights and market economics would not have become ruling principles of free societies had not steam engines and more advanced heat engines provided the energy services that liberate humans from drudgery.

The 18th century had only the Aristotelian notion of *energeia* as a philosophical concept for action or force; entropy was unknown. Adam Smith’s economic world was that of the agrarian society, in which the wealth of nations had been produced for about 10,000 years by the factors capital, labor, and land [22]. Nobody saw that energy is present in so many forms such as light, fire, flowing water, wind, wood, wheat, meat, gun powder, and coal.

Only in the 19th century, when investigating the processes of industrial production, people in the natural sciences and engineering discovered energy and entropy and their pivotal role in these processes. In addition, today we know that our universe started about 14 billion years ago, when all its energy, concentrated in a “point”, exploded in the Big Bang. Since then all entities of the physical world have evolved from energy, while entropy increases.

In the tradition of Adam Smith, conventional neoclassical textbook economics has worked with the production factors capital, labor, and land until these days. The modern concept of capital includes all energy-conversion devices and information processors, and all buildings and installations necessary for their protection and operation. Energy activates the capital stock and labor handles it. Nevertheless, energy is usually not considered to be a factor of production, despite Tryon’s early observation: “Anything as important in industrial life as power deserves more attention than it has yet received from economists ...A theory of production that will really explain how wealth is produced must analyze the contribution of the element energy.” [23] Rather, energy has been and still is considered as just one of the many elements in the basket of natural resources, about which the Nobel laureate in economics R.M. Solow [24] stated: “The world can, in effect get along without natural resources”, adding, however, that “if real output per unit of resource is effectively bounded—cannot exceed some upper limit of productivity which in turn is not far from where we are now—then catastrophe is unavoidable.” Since the useful component exergy of the “natural resource” energy is unavoidably diminished by entropy production in every economic process, real output per unit of energy *is* effectively bounded. Are we, therefore, heading for catastrophe?

### 3.2. Economic Growth, Its Actual Importance, and Neoclassical Theory

Obviously, people fear that industrial free-market economies cannot evolve in stability without the economic growth we have known so far. The growth of gross domestic product (GDP) is considered to be vital for the following reasons. The GDP sums up all salaried economic activities that produce the output of value added within a country. It is measured in monetary units [25]. It includes services that mitigate the damages from accidents, crime, pollution, and other harmful occurrences, such as the abuse of drugs and alcohol, and it excludes the domestic care of people for their children and parents, housekeeping by family members, and community services. Thus, it does not measure the overall well being of a country’s population. This is common knowledge. Nevertheless, the growth of GDP and the growth of the output of economic sectors such as agriculture, industry and services, are of eminent political and social importance, because GDP measures economic activities. People appreciate these activities, notwithstanding their negative side effects, and go where the action is; this drives the rural exodus to the urban centers. One important reason is that economic activities provide jobs, especially when economic growth opens up new fields whose jobs make up for the traditional jobs that are lost to progress in automation. Thus, voters tend to reelect governments that rule in times of growth, and oust the ones they hold responsible for economic recessions. Migrants from less industrialized parts of the world with low GDP/capita risk their lives to get into highly industrialized countries with high GDP/capita. When in 2020 the Covid-19 pandemic drove the world into the deepest recession since the turn of the century, many billions of US Dollars, Yuans, Yens, and Euros were spent by governments, indebting their countries heavily, in order to reestablish economic growth.

The mainstream neoclassical economic theory of production and growth describes the output *Y* of goods and services, which is the gross domestic product or parts thereof, by a function of the inputs of capital *K* and labor *L* [26]. One special type of such a macroeconomic production function, the Cobb-Douglas function of *K* and *L*, had been used by Solow [27,28] in his ground-breaking contribution to the theory of economic growth. He discovered what is called the “Solow residual”. This residual is the big difference between the observed economic growth and the much smaller theoretical growth computed with the empirical data of capital and labor. Solow proposed that “technological progress” is responsible for that part of growth that capital and labor cannot explain. Since then, neoclassical growth theory has been based on production functions $Y_{nc}\left(K,L;t\right)$ with the factor inputs *K* and *L* and a “technological progress” component that depends on time *t* and is determined by minimizing the Solow residual.

### 3.3. Oil-Price Shocks

Between 1973–1975 the oil price on the world market nearly tripled when OPEC “punished the West” for supporting Israel in the Yom-Kippur war. The resulting first oil-price shock interrupted the strong economic growth enjoyed after World War 2 especially by the G7 countries Canada, France, the FRG, Italy, Japan, the United Kingdom, and the USA [29,30]. For instance, within these two years the output slumped by more than 5 percent and by nearly 6 percent in the industrial sectors of the USA and the FRG, respectively; simultaneously, these sectors’ energy use dropped by more than 7 percent in the USA and more than 8 percent in the FRG [31] (p. 200). Another recession was caused by the second oil-price shock between 1979–1981, when the inflation-corrected market price of oil doubled, shooting up to its 20th century maximum, as a consequence of Iraq’s attack on revolutionary Iran and the curb of oil supply from these two major exporters.

The drastic downturns and upswings of economic output and energy use, induced by the oil-price shocks, led economists, in studies such as that by [32,33,34,35,36], to treat energy *E* as a third factor of production on an equal footing with capital *K* and labor *L*, and describe output and its growth by different types of production functions $Y_{nc}\left(K,L,E;t\right)$. In a controversial discussion on whether the first oil-price shock could have been related to the 1973–1975 recession in the USA, the econometrician Denison [37] argued: “Energy gets about 5 percent of the total input weight in the business sector ...the value of primary energy used by nonresidential business can be put at $42 billion in 1975, which was 4.6 percent of a $ 916 nonresidential business national income. ...If ...the weight of energy is 5 percent, a 1 percent reduction in energy consumption with no changes in capital and labor would reduce output by 0.05 percent.”

Denison’s argument is based on the cost-share theorem, one of the pillars of neoclassical growth theory. The cost-share theorem says that a production factor’s economic weight—more precisely: its *output elasticity*, see below—must be equal to the factor’s share in total factor cost. In the G7 countries the cost shares have been roughly 25 percent for capital, 70 percent for labor, and 5 percent for energy. Thus, a 7 percent reduction of energy input, as it was observed for the industrial sector of the USA between 1973 and 1975, should have resulted in a (5 percent)×(7 percent) = 0.35 percent reduction of output. As mentioned above, the actually observed output reduction was more than 5 percent.

Consequently, neoclassical production functions $Y_{nc}\left(K,L,E;t\right)$ with cost-share weighting of K,L,E neither reproduce the recessions and recoveries spurred by the oil-price explosions, nor can they get rid of Solow residuals without neoclassical “technological progress” functions. From the perspective of orthodox economics energy, even if taken into account as a production factor, matters little in economic growth.

This may lead to illusions about easy paths to sustainability: W. Nordhaus received the 2018 Nobel Price in Economics for his research on climate economics. In his book “A Question of Balance. Weighing the Options on Global Warming Policies” [38] (p. 34) he weighs energy’s contribution to production and growth by its cost share [39,40,41,42,43]. Neoclassical growth models are used in integrated assessment models of climate change. Climate activists invoke “the results of science” and demand a rapid and “courageous” exit from the use of oil, gas and coal, which presently satisfy more than 83% of world energy demand. If energy really had an economic weight of only a few percent, a precipitous ban of fossil energy technologies would not cause major economic problems, even if investments in renewables, which are to substitute fossil fuels, should fall way behind. Sufficient were “to wake up politicians” so that they promote the appropriate “technological progress”— whatever that may be.

The dominating role of technological progress “has led to a criticism of the neoclassical model: it is a theory of growth that leaves the main factor in economic growth unexplained”, as the founder of neoclassical growth theory, Robert M. Solow, stated himself [44]. Endogenizing technological progress [45,46,47] does not change the disdain of energy.

The cost-share theorem, which assigns the few-percent weight to energy, results from the conditions for the equilibrium in which an economy is supposed to evolve. These conditions fix the output elasticities of capital, labor and energy in mainstream economics. Roughly speaking, the output elasticity of a production factor gives the percentage of output change when the factor changes by 1 percent [48]. It indicates the economic weight, or productive power, of a production factor.

## 4. Economic Equilibrium and Technological Constraints

Economic growth depends on the preferences of people and technical possibilities. Aspects that matter are:

1 The economic actors choose the *quantities* of factor inputs at time *t* according to the expected demand for output.
2 Neoclassical economics assumes:
  (a) Entrepreneurs select the factor *combinations* that maximize profit or overall welfare; the latter is represented by time-integrated utility. (Preferences that may result from drives for power and grandeur are not considered.) The optimized factor combinations define the equilibrium in which the economy is supposed to evolve.
  (b) All combinations of K,L,E are possible.
3 Engineering experience, however, is that not all factor combinations are possible:
  (a) One cannot feed more energy into the machines of the capital stock than they are designed for. If one would try, the machines would break down. Thus, the degree η(K,L,E) of capital’s capacity utilization cannot exceed 100%.
  (b) The possibility of substituting capital and energy for labor by increasing automation increases with the decreasing mass and volume of information processors. Where the transistor replaces the vacuum tube, it is the density of transistors on a microchip that matters. This density, however, is limited by Joule heating and heat conductivity [49]. Thus, the degree of automation at a given time *t*, ρ(K,L,E), cannot exceed some technological limit $\rho_{T}\left(t\right)$, which trivially, cannot exceed 100%.

The cost-share theorem is invalid, if one or more of the underlying assumptions 1, 2(a), or (2b) are invalid. For the sake of the argument, we do not question 1 and (2a), but focus only on (2b). It turns out to be sufficient to refute the assumption of the general validity of the cost-share theorem by including the constraints 3(a), 3(b) in the optimization of profit/cost, or overall welfare [10,50]. For this, the constraints η(K,L,E)≤1 and $\rho \left(K,L,E\right)\leq \rho_{T}\left(t\right)$ are written in the form of equalities $f_{\eta }\left(K,L,E;t\right)=0$, $f_{\rho }\left(K,L,E;t\right)=0$ with the help of slack variables $K_{\rho },L_{\eta },E_{\eta }$, which are added to K,L,E in the explicit equations for η(K,L,E) and ρ(K,L,E). Optimization subject to the technological constraints in the form of equalities is done by adding these constraints, multiplied by the Lagrange multipliers $\lambda_{\eta }$ and $\lambda_{\rho }$, to the objective function. In the case of profit optimization the objective function is output Y(K,L,E;t) minus total factor cost $p_{K}K+p_{L}L+p_{E}E$, where $p_{K},p_{L},p_{E}$ are the prices per unit of K,L,E. Defining $\left(K,L,E\right)\equiv \left(X_{1},X_{2},X_{3}\right)$ and $\left(p_{K},p_{L},p_{E}\right)\equiv \left(p_{1},p_{2},p_{3}\right)$, and doing the optimization one obtains the equilibrium conditions, which say: The output elasticities of capital $\epsilon_{1}$, labor $\epsilon_{2}$, and energy $\epsilon_{3}$ must be

(1) $$\epsilon_{i}=\frac{X_{i}\left[p_{i}+s_{i}\right]}{\sum_{i=1}^{3}X_{i}\left[p_{i}+s_{i}\right]},\;i=1,2,3\;.$$

Here $s_{i}\equiv -\lambda_{\eta }\frac{\partial f_{\eta }}{\partial X_{i}}-\lambda_{\rho }\frac{\partial f_{\rho }}{\partial X_{i}}\;$ are (generalized) shadow prices, which map the technological constraints into monetary terms. “Generalized” indicates that there are additional “soft” constraints that prevent entrepreneurs from managing the economy in the state where a technological constraint is exactly binding. In such a state, there would be only two instead of three independent variables (K,L,E) and, thus, less freedom to adjust production to changes of demand or factor availability. Between 1960 and 1990 the industrial sector of the FRG evolved on a path in the cost mountain that is high above the neoclassical cost minimum and more or less parallel to the barrier from the binding constraint η(K,L,E)=1 [50]. From experience, entrepreneurs are aware of the technological constraints and steer clear of the barriers formed by them. Only by calling upon “soft constraints” their behavior agrees with the assumption 2(a) of textbook economics. Anyway, decisive is that entrepreneurs know that the assumption 2(b) is wrong. At the energy prices we have known so far, the cost-share theorem is invalid. Optimization of time-integrated utility yields equilibrium conditions such as Equation (1) with somewhat modified $s_{i}$ [50].

If there were no technological constraints, the Lagrange multipliers $\lambda_{\eta }$ and $\lambda_{\rho }$ would be zero, so would be the $s_{i}$, and Equation (1) would be reduced to the cost-share theorem that fixes the output elasticities of neoclassical production functions $Y_{nc}\left(K,L,E;t\right)$: The numerator is the cost of the production factor $X_{i}$, the denominator is the cost of all factors, and the quotient is the cost share.

The technological constraints on factor combinations, ignored in the derivation of the cost-share theorem, drive the wedge between neoclassical growth theory and what really happens in modern economies [51,52].

## 5. Wealth Production and Growth: A Biophysical Analysis

### 5.1. General Outline

The cost-share theorem misleads investigations of economic growth. An alternative biophysical analysis disregards this generally invalid theorem. From neoclassical economics it only adopts the concept of the macroeconomic production function [53,54,55].

Biophysical production functions Y(K,L,E;t) have the independent variables K(t), L(t) and E(t) [56], which the economic actors choose within given technical and legal constraints according to the expected demand for goods and services and the ends they pursue by their economic activities. The Mathematical Appendix, Section 8, presents the basic equations for computing non-neoclassical output elasticities (compatible with (3a) and (3b) of Section 4 above) and the corresponding production functions. The following summarizes that.

Y(K,L,E;t) is a state function of the economic system—just as internal energy and entropy are state functions of thermodynamic systems in (local) equilibrium. As such Y(K,L,E;t) depends only on the actual magnitudes of the variables K(t),L(t),E(t) and not on the path in (KLE)-space along which the system has arrived at them. Consequently, at any fixed time *t*, the growth rate of output, dY/Y, is unequivocally determined by the growth rates of capital dK/K, labor dL/L, and energy dE/E, and the respective output elasticities. In total, the *growth equation* is dY/Y=α·dK/K+β·dL/L+γ·dE/E+δ·dt/Δt, where the last term takes into account a possible explicit time dependenc of *Y*. The second-order mixed derivatives of *Y* with respect to K,L,E must be equal. The resulting three partial differential equations for the output elasticities of capital, α, labor, β, and energy, γ, are coupled by the requirement of “constant returns to scale”, which means that α+β+γ=1 at any fixed time *t* [57]. They have innumerable solutions. The trivial solutions are the constants $\alpha_{0},\beta_{0},\gamma_{0}=1-\alpha_{0}-\beta_{0}$. Non-trivial, i.e., factor-dependent output elasticities are obtained from (asymptotic) boundary conditions that incorporate economic developments such as the one described by the law of diminishing returns. This law, one of the most famous laws of economics [58], says: “At a given state of technology the additional input of a factor, at constant inputs of the other factors, results in an increase of output. Beyond a certain point, however, the additional return from an additional unit of the variable factor will decrease. This decrease is due to the fact that one unit of the increasing factor is combined with less and less quantities of the fixed factors.”

Y(K,L,E;t) abstains from the neoclassical “technological progress function”. It depends explicitly on time, if the technology parameters, which result as integration constants of the differential equations, do so. The parameters are determined by minimizing the deviations of theoretical from empirical growth, subject to the conditions that output elasticities must be non-negative. They change in time when human ideas, inventions and value decisions, which summarily are called “creativity”, change the state of economic systems; δ in the growth equation is the output elasticity of creativity. Creativity, in this context, has positive and negative components such as human rights, the transistor, and to foster agreement, on the one hand, and racism, cheating software in the exhaust control of Diesel cars, and to obstruct cooperation, on the other hand.

### 5.2. Observed and Computed Economic Growth

**Figure 1:**
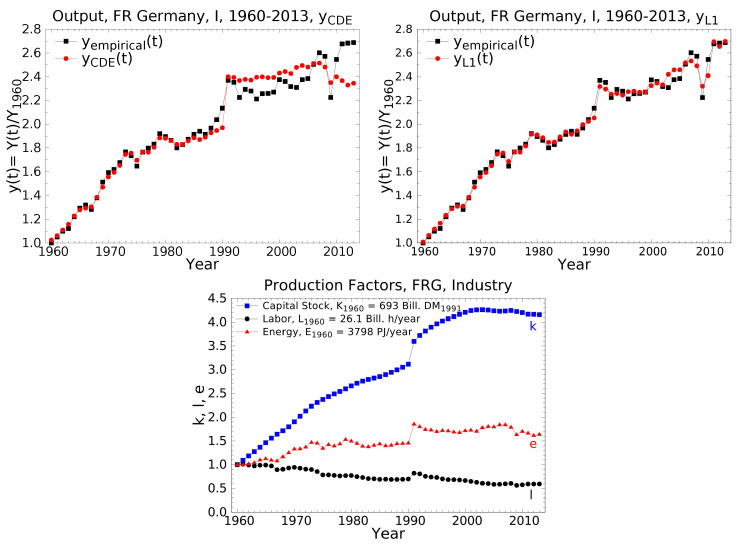
Growth from 1960 to 2013 of the empirical output $y=Y/Y_{1960}$ in the industrial sector of the Federal Republic of Germany (FRG), black squares, and theoretical growth computed with the energy-dependent Cobb-Douglas function, red circles (**left**), and the LinEx function, red circles (**right**). Empirical growth of capital $k=K/K_{0}$, labor $l=L/L_{0}$, and energy $e=E/E_{0}$ (**bottom**). The base year $t_{0}$, to which output and inputs are normalized, is 1960. $Y_{1960}=453.5\times 10^{9}\;{\mathrm{DM}}_{1991}$ [59].

Biophysical production functions have been applied to economic growth in highly industrialized countries since 1982 [31]. Recent results for the USA and the FRG from 1960-2013 are reported by Lindenberger et al. [59]. Figure 1 is an example from the sector “Industries” (I) of the FRG. There, the strongest variations of empirical output and inputs occurred. Since 1990 these variations have been influenced by the only territorial enlargement of a major industrial country after World War 2. They test the sensitivity of production functions to technological and structural changes, and political and psychological perturbations as well. Two production functions were utilized for the reproduction of the observed growth: On the one hand the energy-dependent Cobb-Douglas function $Y_{CDE}$, Equation (7), whose constant output elasticities turn out to be $\alpha_{0}=0.41,\beta_{0}=0.06,\gamma_{0}=0.53$, and on the other hand the LinEx function $Y_{L1}$, Equation (9), with *factor-dependent* output elasticities, whose *time-averages* result to be $\bar{\alpha }=0.28$, $\bar{\beta }=0.08$, $\bar{\gamma }=0.64$, and $\bar{\delta }=0.13$.

$Y_{L1}$ is the simplest production function of the LinEx-function family, whose members depend *lin*early on one factor, here *E*, and *ex*ponentially on the quotients of the other factors. More complicated LinEx functions are given in [10,59]. They are all special forms of the general linearly homogeneous, twice differentiable, energy-dependent production functions that solve the growth Equation (2). The latter are shown by Equations (10)–(12) of the Mathematical Appendix, Section 8.

Noteworthy features of empirical and theoretical growth in Figure 1 are:

1. Between 1960 and 1990 the energy-dependent Cobb-Douglas function with its constant output elasticities reproduces observed growth nearly as well as the LinEx function with its factor-dependent output elasticities. After 1990 LinEx is much better. (Its adjusted coefficient of determination is ${\bar{R}}^{2}=0.99$ and the Durbin-Watson coefficient is $d_{w}=1.75$; the statistically best values are 1 and 2, respectively.) Both the time-averaged LinEx and the constant Cobb-Douglas output elasticities are for energy much larger and for labor much smaller than these factors’ cost shares. Please note that also the sum of the time-averaged LinEx output elasticities that are related to routine and “creative” activities of humans, $\bar{\beta }+\bar{\delta }$, stays well below energy’s output elasticity $\bar{\gamma }$.
2. Creativity’s component “value decisions” was activated, when, unexpectedly, the winners of World War 2 agreed to let divided Germany reunite in 1990: Factor inputs and output increase abruptly in 1990. (The LinEx technology parameter “energy demand of the capital stock” does the same [59].)
3. The bidirectional causality that rules the coupling of energy and economic growth shows in the four economic recessions and recoveries and the simultaneous downs and ups of the energy input. Two of them were caused by supply and two by demand, and three were enhanced by feedbacks between the two. The supply side triggered the first and the second oil-price shock 1973–1975 and 1979–1981: The oil-price explosions, caused by OPEC, made investors worry about shortages of power fuel for their machines so that they substantially reduced investments. A demand-side element amplified the shocks: Part of the consumers’ buying power had been skimmed by the oil producers. Thus, consumers demanded less goods and services. To satisfy the reduced demand from investors and consumers less energy was needed for production. When the oil-price stopped shooting up, the shocks subsided, and growth of output and energy consumption restarted. Demand-side triggering occurred, when between 1965 and 1966 the ruling conservative-liberal coalition of the FRG became unstable. The resulting economic uncertainties led to reductions of investment, consumption and energy use. Then, for the first time after WW 2, the social democrats became part of the federal government. The new coalition restored confidence in the country, ended the economic crisis, and with increasing demand for goods and services energy consumption rose again. Similarly, the *global* financial crisis 2007–09 was due to a demand-side trigger: After the global breakdown of stock markets, demand for goods and services slumped, machines went idle and did not need energy, until banks were saved by the taxpayers’ money so that confidence in the economy came back, and demand for output and energy rose. On the other hand, the burst of the US mortgage bubble, which caused the initial crash of the US stock market, is related by Murray and King [60] to a supply-side effect: Before 2007, the oil price had risen to more than 100 US${\$}_{2014}$/barrel. The higly indebted homeowners in the American suburbs were confronted with exploding costs for commuting to their jobs and could not pay their mortgage interests any more.
4. The overall growth of output follows the empirical growth of the capital stock. The latter’s flattening and even decrease reflects outsourcing in German industry. The share of the industrial sector in the GDP of the FRG decreased from 51.7% in 1970 to 39.6% in 1992 to 27.1% in 2009 [10] (p. 193) Especially, energy-intensive and polluting industries have been shifted to developing countries and emerging economies. This has stopped the growth of the industrial capital stock and contributes substantially to the reduction of German energy consumption and CO${}_{2}$-emissions. The decrease of labor input, which is also observed in the total economy of the FRG [10,59], is due to outsourcing and increasing automation.

Growth of output with its ups and downs in the total economies of the FRG and the USA from 1960 to 2013 is also well reproduced by the LinEx function and its factor-dependent output elasticities [59]. Again, the time-averaged output elasticities turn out to be for energy much larger and for labor much smaller than those factors’ cost shares. Manrique-Dias and Lemus-Polonia [61] computed economic growth in Colombia from 1925 to 1997. The LinEx function, with “electricity consumption” as the energy variable *E*, reproduces the empirical growth of Colombian GDP satisfactorily. The output elasticities have time averages similar to the ones of the total economy of the FRG and patterns of temporal variations that somehow resemble those of the total US economy.

Using “useful work” instead of primary energy in a formally modified LinEx function Ayres and Warr [6] computed economic growth in the USA and Japan from 1900 to 2005 (excluding 1941–1948) in good agreement with observed growth. Useful work is the exergy that works directly from the machines on materials plus the physical work performed by animals. The data on it in [62] incorporate efficiency improvements of the energy-converting systems. The magnitudes of the output elasticities that result from this analysis contradict the cost-share theorem, too. This analysis stimulated more research on “exergy economics”, such as [63]. Earlier studies on the pivotal role of energy in economic growth led Hall et al. to emphasize “the need to reintegrate the natural laws with economics” [64].

Computation of future economic growth could be done via scenarios concerning entrepreneurial choices of capital, labor, and energy, in which the crises ahead will challenge creativity. For this, models such as the HARMONEY model [65], a long-term dynamic growth model that endogenously links biophysical and economic variables in a stock-flow consistent manner, may be useful. Furthermore, production functions with output elasticities that take into account the impact of emission mitigation [21], may also serve as analytical tools. Consistent data on capital, labor, and energy in different sectors of the economy will be important. Studies on past growth have shown that inconsistent data lead to breakdowns of production-function estimations. The sources and structures of the data used in our most recent study on energy and economic growth are documented in [59] (Appendix 3).

## 6. Crises and Creativity

The strong coupling between energy and economic growth via bidirectional causality has shown especially in times of crises. There have been and will be crises related to politics and markets, and crises involving natural challenges and human responses.

### 6.1. Politics and Markets

Initially, the two economic recessions in 1973–1975 and 1979–1981 were called “energy crises”. However, “oil-price shocks” better indicates the psychology involved. After the oil-price had settled on its 1975 level, the shock wore off, and output resumed growth despite the tripled oil price. The cost share of all energy carriers in total factor cost was still much lower than energy’s productive power. Even the next oil-price explosion in 1979 did not change this. However, it caused the second shock and the resulting recession. After the Iraq-Iran war the oil-price collapsed [66], the economic actors in the market economies relaxed, and growth restarted from about the 1978 level. To the recovery also contributed the development of nuclear energy, the discovery of new, non-OPEC oil fields, and the reinvestment of petro dollars in the G7 countries. Here, the solutions to the crises came from the easing of tensions in international politics and markets, the opening up of new energy sources, and the self-interest of the owners of surplus petro dollars.

The 1965–1967 crisis in the FRG ended with the recovery of political stability. The 2007–2009 financial and economic crisis was overcome when central banks, especially the FED and the ECB, did “Whatever it may take” to help tattered firms with direct or indirect subsidies and battered states with bond purchases and cuts of interest rates. This contributed to the mounting public debt and losses on bank deposits.

On May 5, 2020, the Federal Constitutional Court of the FRG, after several years of legal deliberations, ruled that the Public Sector Purchase Program (PSPP) of the ECB has violated the principle of comparativeness insofar as government bonds were also purchased with the aim to keep the inflation rate close to 2%. According to the estimation of the ECB, if inflation were less, deflation would hamper economic growth. Actual inflation had been below the 2% level, because the price of a barrel of crude oil had dropped from nearly 120 US$${}_{2014}$ in 2012 to less than 40 US$${}_{2014}$ in 2014. Since then it had been fluctuating somewhat until the end of the decade. The prices of most other consumption goods, however, had risen so much that consumers did *not* delay spending in expectation of deflation. However, obviously, the ECB considers energy as just another commodity. A better understanding of the impact of energy and its price on economic growth by decision makers would have avoided that, in the worst case, the Central Bank of Germany will be forced to withdraw from the ECB.

Eichhorn and Solte analyzed the global financial system. They point out that in 2008, new indebtedness of public sector entities world wide was higher than global savings performance, and that global securitized assets exceeded the global stock of central bank money—the only legal tender—by a factor of 50. In the 40 years before, global financial and tangible assets grew more rapidly than global value added (GDP). If the past trends of interest and return on investment (ROI) were to continue in the future, by the year 2030 all of global GDP would be necessary to service the accumulated debts. Nothing would be left to pay employees. [67] (pp. 190–193).

In the long run the most dangerous crises in the field of politics and markets may originate from the inequalities of wealth distribution on national and international scales and their consequences of civic unrest and international conflicts. The inequality of income distribution *within* several OECD countries has been measured by the Luxembourg Income Study [68] by means of the Gini coefficients *G*, 0≤G≤1, which result from those countries’ Lorentz curves [10] (p. 185). The larger *G* the higher the inequality. According to the study, in the mid-1980s *G* was close to 20% for Finland, Sweden and Norway, and it exceeded 30% for Switzerland, Ireland, and the USA. The *global* inequality of *wealth* distribution in 2005 is indicated by the shares of the rich and the poor in world’s private consumption of *goods and services* per wealth/poverty level [10] (p. 232f), [69]. The wealthiest 10 percent of world’s population had a share of 59% of world’s private consumption, whereas the share of the world’s poorest 50 percent was just 7.2%. By 2005 approximately half the world’s population lived in cities and towns, where one out of three urban dwellers (approximately 1 billion people) was living in slum conditions. In developing countries some 2.5 billion humans were forced to rely on biomass—fuelwood, charcoal and animal dung—to meet their energy needs for cooking; this sort of biomass is usually not included in the international energy statistics.

Lawrence, Liu, and Yakovenko [70] analyze the global probability distribution of *energy* consumption per capita around the world from 1980–2010. This impressively complements the statistics on global wealth distribution. Their Lorentz curves “Fraction of World Energy Consumption” vs. “Fraction of World Population” involve the USA, USSR/Russia, France, the UK, China, Brasil, and India, and correspond to Gini coefficients G of 0.66 in 1980, 0.64 in 1990, 0.62 in 2000, and 0.55 in 2010. Thus, within 30 years the global inequality of *energy* consumption per capita has decreased [71]. However, still 70 percent of the world’s population in developing and emerging economies had a fraction of less than 40 percent of world energy consumption in 2010. The remaining more than 60 percent of energy consumption went to the 30 percent of world population in the industrialized countries. Many of the latter belong to the wealthiest ones, with high shares of private consumption and small inequalities of income distribution, i.e., Gini coefficients not much above 30%, as mentioned above.

The statistical findings on the distributions of wealth and energy consumption support the econometric findings that energy is an important factor in the production of wealth.

Since the 1960s, the programs of development assistance have aimed at fostering the well being of the people in the developing countries by (a) increasing their countries’ GDP and (b) by reducing the inequalities of internal wealth distribution. Aim (a) has been reached to some extent by promoting industrialization and energy consumption world wide. Progress in reaching aim (b) has been slow. It may be advanced by appropriate energy taxation and/or an international agreement on preventing the flight of capital from the developing countries to the highly industrialized countries. However, the threats from emissions and climate change because of entropy production may endanger even further progress towards aim (a). In addition, even more disquieting, Lawrence, Liu and Yakovenko deduce from the principle of maximum entropy production that one may never achieve a less unequal distribution of global energy consumption than the one represented by the Lorentz curve with a Gini coefficient of 0.5 in [70] (Figure 3). The expectation that this may also lead to a corresponding stable global inequality in the distribution of CO${}_{2}$-emissions has been recently confirmed [72]. Are we approaching a stagnation in which “the world is likely to stay put in the present state of global inequality”, because “human development for centuries was driven by geographic expansion, but this era is over” [70] (p. 5573)?

Space industrialization with solar power satellites, discussed below, may provide a way out of stagnation. It may also provide the last resort (for some), if outbreaks of supervolcanoes with high extinction potential that lurk below the Yellowstone Park and the Phlegraean Fields materialize.

### 6.2. Natural Challenges and Human Responses

1. Risk assessments of energy resources and technologies
  On March 11, 2011, one of the worst earthquakes in the history of Japan, and the tsunami it caused, destroyed the Fukushima 1 nuclear power facility erected right on the Pacific ring of fire on the east coast of Japan. The earthquake severed the connection to the electricity grid and the Tsunami inundated the emergency generators of four reactor blocks, built just 10 m above sea level. The emergency shutdown of three reactors worked well. A fourth reactor had been deactivated, and its nuclear fuel rods were cooled in the fuel pit. Because of the lack of cooling, the nuclear waste heat from β-decay could not be removed, three reactors suffered core meltdowns, and the fourth exploded, most likely because of oxygenhydrogen formation in the hall containing the fuel pit [73,74]. On the whole, the radioactive emissions caused by the Fukushima accident were 10 to 20% of the catastrophe in Chernobyl, where a graphite-moderated reactor blew up in a failed safety experiment. Prior incidents in Japanese nuclear power stations in 2005 and 2007 had already shown that their design, adopted from reactors in the USA, had not been modified properly to meet the known risks that exist in Japan. One had decided to accept them.
  In the 2009 electoral campaign for the German Bundestag, the ruling coalition under chancellor Dr. Merkel promised that it would extend the legal operation time for the German water-moderated nuclear reactors by up to 14 years. Otherwise, it was said, Germany would not be able to meet her aims of reducing CO${}_{2}$-emissions. The coalition was reelected with a comfortable majority, and the parliament passed the law on the operation-time extension. Right after the Fukushima catastrophe, in a U-turn of German energy policy called “Energiewende”, the government of Dr. Merkel proposed the total exit from nuclear power, and the parliament decided it. Eight reactors were shut off right away, and of the remaining nine the last one is scheduled to cease operation in 2022. In a mix-up of “known risk” and “residual risk” Dr. Merkel told the public that the reason for the U-turn was the underestimation of the residual risk of German nuclear reactors. Actually, the probability that an accident as in Fukushima would occur in Germany is equal to the probability of a heavy earthquake in Germany *and* that a tsunami destroys the emergency generators of four nuclear power plants in the country.
  Germany claims a cutting edge in climate protection [75,76]. Experience will show, how she lives up to that claim. After the banning of nuclear power without changing the German road map for reducing CO${}_{2}$-emissions, renewable energies must fill the gap in electricity generation that would open up, if coal and lignite power plants would be abolished as planned originally. Success or failure of renewable energies will decide, whether, in the end, the “Energiewende” will turn out as either a positive or a negative element of creativity. The uncertainty results from the phenomenon of *size-dependent risk perception*, which is a fundamental problem faced by energy policy everywhere: When an energy source contributes noticeably to the energy supply of an economy, its inevitable side effects will affect the environment. If people notice them, there will be protests, often pursuant to the NIMBY (Not In My BackYard) principle. Side effects that go unnoticed for some time, may become big problems in the future.
  Renewables are an example. In 2018 they contributed just 4% to global primary energy consumption [77]. In Germany, their total share in primary energy was about 13%, with the shares of biomass, wind, and photovoltaics being 7.1%, 2.8%, and 1.1%, respectively [78].
  (a) Biomass dominates. It is a storage of solar energy and well accepted by the population. However, the National Academy of Sciences (Leopoldina) points out that biomass has a bad *Energy Return on (Energy) Investment* (EROI) [79], mostly below 3, that its production threatens biodiversity, damages soil quality, pollutes ground water, rivers and lakes, and that financially it has the highest price per saved ton of CO${}_{2}$ [80].
  (b) Wind power is heavily attacked by civic movements. The given reasons are: Onshore wind turbines make noise, cast whirling shadows, kill birds, and spoil the landscape. The high-voltage transmission lines that shall carry electric power from offshore wind parks in the wind-rich north of Germany to southern Germany are rejected for esthetic reasons and their land requirements. The protesters ignore that the specific total life-cycle CO${}_{2}$-emissions of wind parks are only 10–20 g CO${}_{2}$ per kilowatt-hour of electric energy—similar to those of nuclear power plants—and the lowest of all renewables.
  (c) Photovoltaics (PV), whose specific total life-cycle CO${}_{2}$-emissions range from 70 to 150 g CO${}_{2}$ per kilowatt-hour, is still well accepted. To keep it that way the government has tried to limit the payments of the electricity consumers to the providers of PV power to 10–11 billion Euros annually [21]. Looking into the future, GreenMatch, “a comprehensive guide designed to help you navigate the transition to renewable energy” [81] points out the need to recycle PV panels when their life cycle ends: “If recycling processes were not put in place, there would be 60 million tons of PV panels waste lying in landfills by the year 2050; since all PV cells contain certain amounts of toxic substances that would truly become a not-so-sustainable way of sourcing energy.” GreenMatch estimates the amount of solar panel waste (in tons) to be for (a) the USA in 2016: 6500 t, 2030: 400,000 t, 2050: 7500,000 t, (b) Germany in 2016: 3500 t, 2030: 400,000 t, 2050: 4300,000 t, (c) Saudi Arabia in 2016: 200 t, 2030: 3500 t, 2050: 450,000 t. The energy requirements for recycling these quantities of PV waste, and the associated emissions and cost, remain to be estimated.
2. Pandemics
  The economic instruments to fight the 2007-09 financial and economic crisis have been reactivated in the Corona crisis that started with the outbreak of the Covid-19 pandemic in Wuhan, China, by the end of 2019. Since then, severe constraints on the interaction between people have been imposed by governments all over the world and successively strangulated commercial, artistic and educational activities. Employment slumped. This has dwarfed the demand for many goods and services, their production ceased, and so did the demand for energy. Occasionally, the oil price even became negative, when the producers of conventional oil and the US-producers of oil from fracking would not or could not reduce oil production, while all the oil-storage facilities were filled up. As in the 2007-09 crisis, the actions of governments and central banks to stabilize economies—and this time also public health—boost public debt. To complicate things, health and environmental protection must be balanced with economic and social losses. The G7-countries are especially vulnerable to the constraints imposed on personal interactions in times of pandemics such as Corona, because the share of their service sectors in both employment and GDP has been roughly 70% since the turn of the century [10] (p. 193)
3. Limits to growth in the biosphere
  Two ways of dealing with the thermodynamic limits to industrial growth in the biosphere are (a) to adapt to them via transition to a post-growth economy, and (b) to surmount them via space industrialization.
  (a) Niko Paech [82] proposes that the highly industrialized societies adapt to the ecological constraints that exist on the surface of Earth, by changing lifestyles and patterns of supply. This implies a cultural change to sufficiency, and it involves three levels: local subsistence, a regional economy, and a significantly shriveled residual industry. To cushion the reductive transition socially, especially to achieve full employment, a reallocation of the reduced time for gainful occupation will be necessary. 20 h of conventional labor, which are the basis for a reduced monetary income, can be complemented by another 20 h of working for self-sufficiency. Indigenous production, extension of service life, collective use of capital goods etc. will help to continue the use of modern consumption functions and simultaneously realize a higher degree of economic autonomy. Firms can support this development by contributing in many ways to satisfying needs without actually producing new goods.
    Contrary to happy “green” utopias, Niko Paech’s transition scheme to a post-growth economy is sober and realistic. Sober, because it clearly tells people what drastic changes of personal behavior will be necessary. Realistic, because it combines well-known elements of the stationary societies, in which human civilizations have evolved during the last 10,000 years, with the production facilities of the industrial age, whose growth dynamics now threatens the stability of the biosphere. The problem is that the stationary societies of the past had rigid social structures with little social mobility. Traveling for pleasure was unusual.
    Nieto et al. [83] applied an ecological macroeconomics model to the Energy Roadmap 2050 (ER2050) of the European Union; this roadmap has ambitious emission-mitigation targetes, to be achieved by reducing energy use and a transition to renewables. Their “results show that GDP growth and employment creation may be halted due to energy scarcity if the ER2050 targets are met even considering great energy efficiency gains. In addition, the renewables share would increase enough to reduce the energy imports dependency, but not sufficiently to meet the emission targets. Only a Post-Growth scenario would be able to meet the climate goals and maintain the level of employment.”
    In the present Covid-19 pandemic, people suffer from and complain about constraints on professional and leisure activities, many of which are linked to industrialization. Perhaps we can learn from the pandemic how well modern humans will accept the changes of lifestyle, and of the production and distribution of wealth, which may be necessary for adaptation to the stationary society of a Post-Growth age.
  (b) Ancient and modern history tell tales of expansion, when resources become scarce and pioneers, full of vigor and zest for action, set out for new territories with wide-stretching frontiers. The scarce resource of the past was fertile land, whose plants capture the solar energy needed by humans and animals.
    Presently, scarce is the space that, without harmful side effects, can absorb the emissions of industrial energy conversion. However, vast is the space beyond the biosphere. For more than four billion years it has absorbed all heat and particle emissions that accompany the production of life-giving sunlight by nuclear fusion in the core of the Sun. Being aware of this, since the early 1970s, and for about two decades, young, middle-aged, and old scientists from many disciplines had tried to promote a grand design of using extraterrestrial resources to surmount the limits to growth. It implies delivery of clean electric energy to Earth via solar power satellites (SPS) and the production of them in space-manufacturing facilities by people who live in large habitats that orbit around the Lagrange libration point L5. The sources of most of the required energy and materials would be the Sun and the Moon.
    Peter E. Glaser from Arthur D. Little, Inc., proposed and patented solar power satellites [84,85,86]. They are to be stationed in geosynchronous Earth orbit, always above the same point on the equator at a maximum distance of 35,785 km. They convert sunlight into electric energy, either by photovoltaic cells or by solar thermal dynamic systems. Klystrons convert the electric energy into microwaves of about 3-GHz frequency, which are beamed from a transmitting antenna, diameter 1km, of the satellite to a receiving antenna on Earth, diameter 10 km. There, the microwave energy is reconverted into electricity, which is fed into the public grid. Typical generating capacities of SPS are 5000–10,000 MW at bus bar on Earth. The total mass of a SPS is between 34,000 and 86,000 t. This and more, e.g., Boeing’s SPS design and NASA’s system studies, is documented in [87,88,89].
    The big problem is transportation of people and initially required materials to low Earth orbit via chemical rockets. After the catastrophes of the Challenger and Columbia space shuttles in 1986 and 2003, the USA terminated the Space Shuttle Program in 2011. Since then, for the transportation of US astronauts to the International Space Station (ISS), the USA have bought seats in the Russian Sojus rockets. Finally, US billionaires are coming to the rescue of the US space program. For instance, Elon Musk’s commercial “Space X” enterprise builds reusable rockets and space capsules for the transportation of freight and astronauts. There are plans to return to the Moon and go to Mars [90]. China is vigorously pursuing such plans, too. Once on the Moon, one could resuscitate the grand scheme of Princeton physics professor Gerard K. O’Neill to catapult Moon-material via electromagnetic mass drivers [10] (p. 88f) to catchers in the libration point L2 and transfer it to space-manufacturing facilities. There, SPS and habitats for the people who construct and maintain them, would be built [91,92,93,94]. Outside the gravitational abysses of planets, traveling large distances requires little energy. O’Neill’s scheme to open up “The High Frontier” of space for humanity led Representative Olin Teague to present the “House Concurrent Resolution 451” [93] to the 95 Congress of the USA on 15 December 1977. It was referred to the Committee on Science and Technology and closes with the words: “Whereas the ‘High Frontier’ of Space does provide valid opportunities whereby we can conserve and enhance humanity’s existence on Earth, including but not limited to such social and economic benefits as greater employment, a cleaner environment, new energy sources, new knowledge and understanding...: Now, therefore be it *Resolved by the House of Representatives (the Senate concurring)* ...: the Office for Technology Assessment specifically is requested to organize and manage a thorough study and analysis to determine the feasibility, potential consequences, advantages and disadvantages of developing as a national goal for the year 2000 the first manned structures in space for the conversion of solar energy and other extraterrestrial resources to the peaceable and practical use of human beings everywhere.”
  On 9 November 1989 the Berlin Wall came down. Thereafter, the Iron Curtain dissolved, and the Cold War with its threat of humankind’s self-destruction ended. However, the competitive pursuit of power, ingrained in human nature, continues. In the 20th century, those who ruled the seas and the air dominated the world. In the 21st century, the powers in space will become the masters of Earth. If the colonization of space is forgone, humans must tame their competitive drives and dedicate their resources and creativity to dealing with the thermodyamic limits to growth. In either case, cooperation between individuals and nations in strict observation of the constraints from human and natural laws will be needed more than ever.

## 7. Summary and Conclusions

The laws of physics on energy conversion and entropy production have stimulated economic growth analyses via biophysical production functions of capital, labor, and energy. They are solutions of a set of differential equations and their asymptotic boundary conditions. Three efficiency-related integration constants may become time dependent when human ideas, inventions and value decisions, in short: “creativity”, change the state of the economy. The biophysical production functions and their estimation disregard the cost-share theorem of neoclassical economics, because it is flawed: When optimizing profit or overall welfare, one must take into account the technological constraints on factor combinations; these, however, were ignored in the neoclassical derivation of the cost-share theorem. This theorem, which assigns only a small economic weight to energy, is invalid at the low energy prices we have known so far.

The biophysical analyses well reproduce the observed economic growth and its crises in major industrial countries during more than 50 years. The resulting economic weights (output elasticities) are for energy much larger and for labor much smaller than these factors’ shares in total factor cost. While creativity is qualitatively decisive in the long run, its quantitative contribution to growth is much smaller than the one that neoclassical growth theory assigns to “technological progress”.

In highly industrialized countries the growth of gross domestic product, and parts thereof, follows the growth of the capital stock. Despite the outsourcing of energy-intensive industries and the shifting of production to the service sector, in times of economic recessions and recoveries economic output and energy consumption decrease and increase simultaneously. This shows the bidirectional causality between energy and economic growth, which follows from energy’s economic role of activating the capital stock.

Since energy conversion is a powerful driver of industrial growth, and since it is inevitably coupled to emissions of particles and heat via the entropy law, the stability of the biosphere is threatened. Understanding the production and growth of wealth, and careful assessments of the risks and opportunities involved with energy sources and the technologies of their use, are necessary for successful adaptation to the ecological constraints on growth. Experiences from past crises should be remembered. Once the feasible options for adequate technological and social changes are identified, people will hopefully follow creative leadership on the most promising path of future economic evolution.

## 8. Mathematical Appendix

The total differential of the production function Y(K,L,E;t), divided by the production function itself, yields the growth equation:

(2) $$\frac{dY}{Y}=\alpha \frac{dK}{K}+\beta \frac{dL}{L}+\gamma \frac{dE}{E}+\delta \frac{dt}{\Delta t},\;\;\delta \equiv \frac{\Delta t}{Y}\frac{\partial Y}{\partial t}\;,$$

where

(3) $$\alpha \equiv \frac{K}{Y}\frac{\partial Y}{\partial K},\;\beta \equiv \frac{L}{Y}\frac{\partial Y}{\partial L},\;\gamma \equiv \frac{E}{Y}\frac{\partial Y}{\partial E}\;$$

are the *output elasticities* (productive powers) of capital, labor, and energy, respectively. δ in Equation (2) results formally from the explicit time dependence of the production function via time-dependent technology parameters and economically from the influences of human ideas, inventions and value decisions on economic evolution. These influences are summarized by the concept of *creativity*; $\Delta t=t-t_{0}$, where $t_{0}$ is an arbitrary base year with the factor inputs $K_{0},L_{0},E_{0}$.

Since Y(K,L,E;t) is a state function, its second-order mixed derivatives with respect to K,L,E must be equal. Calculating these derivatives from the growth Equation (2) one obtains the integrability conditions

(4) $$L\frac{\partial \alpha }{\partial L}=K\frac{\partial \beta }{\partial K},\;E\frac{\partial \beta }{\partial E}=L\frac{\partial \gamma }{\partial L},\;K\frac{\partial \gamma }{\partial K}=E\frac{\partial \alpha }{\partial E}.$$

The growth equation is integrated at a fixed time *t*, when the production factors are K=K(t),L=L(t),E=E(t). The integral of the left-hand side from $Y_{0}\left(t\right)$ to Y(K,L,E;t) is $\ln\frac{Y(K,L,E;t)}{Y_{0}\left(t\right)}$. It is equal to the integral of the right-hand side:

(5) $$F{\left(K,L,E\right)}_{t}\equiv \int_{P_{0}}^{P}\left[\alpha \frac{dK}{K}+\beta \frac{dL}{L}+\gamma \frac{dE}{E}\right]ds\;.$$

This integral can be evaluated along any convenient path *s* in factor space from an initial point $P_{0}$ at $\left(K_{0},L_{0},E_{0}\right)$ to the final point *P* at (K(t),L(t),E(t)). With $\ln\frac{Y(K,L,E;t)}{Y_{0}\left(t\right)}=F{\left(K,L,E\right)}_{t}$ the production function becomes

(6) $$Y\left(K,L,E;t\right)=Y_{0}\left(t\right)\exp\left\{F{\left(K,L,E\right)}_{t}\right\}.$$

The integration constant $Y_{0}\left(t\right)$ is the monetary value of the basket of goods services at time *t*, if it were produced by the factors $K_{0},L_{0}$, and $E_{0}$. If creativity were dormant during the time interval $t-t_{0}$, $Y_{0}\left(t\right)$ would also be equal to the output at time $t_{0}$.

The partial differential Equations (4) turn into three coupled partial differential equations for α and β, if one uses γ=1−α−β according to “constant returns to scale”, as substantiated in Section 5.1.

The trivial solutions of these differential equations are the *constant* output elasticities $\alpha_{0},\beta_{0}$ and $\gamma_{0}=1-\alpha_{0}-\beta_{0}$. With them, and Equations (5) and (6), one obtains

(7) $$Y_{CDE}\left(K,L,E;t\right)=Y_{0}\left(t\right){\left(\frac{K}{K_{0}}\right)}^{\alpha_{0}}{\left(\frac{L}{L_{0}}\right)}^{\beta_{0}}{\left(\frac{E}{E_{0}}\right)}^{1-\alpha_{0}-\beta_{0}}.$$

This is the simplest energy-dependent production function. It bears the names of Cobb and Douglas, who had constructed a function of such structure, but *without* energy, in the 1920s. The Cobb-Douglas function of capital and labor has been and still is frequently used in neoclassical economics. The simplest non-trivial solutions are the *factor dependent* output elasticities

(8) $$\alpha =a\frac{\left(L/L_{0}+E/E_{0}\right)}{K/K_{0}},\;\beta =a\left(c\frac{L/L_{0}}{E/E_{0}}-\frac{L/L_{0}}{K/K_{0}}\right),\;\gamma =1-a\frac{E/E_{0}}{K/K_{0}}-ac\frac{L/L_{0}}{E/E_{0}}.$$

The output elasticity of capital, α, satisfies in the simplest way the law of diminishing returns, β is the simplest solution of the partial differential equation that couples α and β, and γ results from constant returns to scale. (More details on the factor dependencies of α and β in view of the capital stock’s degrees of utilization and automation are given in [10,31].) With them and Equations (5) and (6) one obtains the (first) LinEx function

(9) $$Y_{L1}\left(K,L,E;t\right)=Y_{0}\left(t\right)\frac{E}{E_{0}}\exp\left[a\left(2-\frac{L/L_{0}+E/E_{0}}{K/K_{0}}\right)+ac\left(\frac{L/L_{0}}{E/E_{0}}-1\right)\right].$$

The parameter *c* measures the energy demand of the fully utilized capital stock, and the parameter *a* is a measure of capital’s effectiveness in producing output when activated by energy and handled by labor. The technology parameters *a* and *c* become time dependent, when creativity is active. They, and $Y_{0}\left(t\right)$, are determined by minimizing the sum of squared errors over all observation times $t_{i}$, i.e., $SSE=\Sigma_{i}{\left|Y_{empirical}\left(t_{i}\right)-Y_{theoretical}\left(t_{i}\right)\right|}^{2}$, subject to the constraints α≥0,β≥0,γ≥0; the Levenberg-Marquardt algorithm in combination with the Ceres Solver statistics program was applied to this problem of non-linear optimization in [59] (p. 9).

The most general production function, in which the output elasticity of energy is known from γ=1−α−β, and α and β have to be determined from their three coupled partial differential equations and appropriate asymptotic boundary conditions, is

(10) $$Y=EF\left(\frac{L}{K},\frac{E}{K}\right).$$

Production functions of the general type (10), especially the LinEx function (9), have been used to analyze economic growth in [6,10,31,50,59,61,64], and references therein [95].

The most general production function, in which the output elasticity of labor is known from β=1−α−γ, whereas α and γ have to be determined from their three coupled partial differential equations and appropriate asymptotic boundary conditions, is

(11) $$Y=LG\left(\frac{L}{K},\frac{E}{K}\right).$$

A special, LinEx-type function of this form has been used to describe the growth of service industries, which also include increasingly digitized processes, e.g., in banking, insurance, and public administration [96]. Another type may be interpreted as describing the evolution of economies in an early state of industrialization.

Finally, the most general production function, in which the output elasticity of capital is known from α=1−β−γ, and β and γ must be determined from their three coupled partial differential equations and appropriate asymptotic boundary conditions, is

(12) $$Y=KH\left(\frac{L}{K},\frac{E}{K}\right).$$

The simplest LinEx-type production function of this form may describe a future state of total digitization.

F,G,H are twice differentiable with respect to L/K and E/K.
